# Quantitative trait loci for tuber blackspot bruise and enzymatic discoloration susceptibility in diploid potato

**DOI:** 10.1007/s00438-017-1387-0

**Published:** 2017-10-27

**Authors:** Agnieszka Hara-Skrzypiec, J. Śliwka, H. Jakuczun, E. Zimnoch-Guzowska

**Affiliations:** Plant Breeding and Acclimatization Institute, National Research Institute, Młochów, Platanowa 19, 05-831 Młochów, Poland

**Keywords:** Bruising, Enzymatic discoloration, QTL analysis

## Abstract

Tuber tissue discolorations caused by impact (blackspot bruising) and enzymatic discoloration (ED) after tuber cutting are crucial quality traits of the cultivated potato. To understand the complex genetics of the traits, quantitative trait locus (QTL) analysis using diploid mapping population and diversity array technology (DArT) markers was performed. The phenotypic assessment included the complex evaluation of blackspot bruising susceptibility through two methods: rotating drum (*B*_RD_) and falling bolt (*B*_FB_) in combination with the evaluation of enzymatic discoloration. Because of observed in-practice relationship between bruising susceptibility and tuber starch content (TSC), analysis of starch content-corrected bruising susceptibility (SCB) was performed. QTLs for bruising were detected on chromosomes I, V with both test methods. The rotating drum method enabled the detection of additional QTLs on chromosomes VIII and XII. Analysis of SCB enabled the identification of the major QTL on chromosome V and two weaker QTLs on chromosomes VIII and XII, independently of starch content. The QTL for bruising detected on chromosome I overlapped with the most significant QTL for tuber starch content. This QTL was not significant for starch content-corrected bruising susceptibility, and the effect of the QTL on chromosome V was enhanced for this trait. The QTL analysis of ED revealed the contribution of seven QTLs for the trait, located on six chromosomes, including these detected for the first time: a major locus on chromosome V and minor QTLs on chromosomes VII and X, which were specific for the trait. The QTL for ED on chromosome VIII was co-localized with the marker for polyphenol oxidase (*POT32*). The phenotypic correlation between bruising and ED was confirmed in QTL analyses of both traits, and the QTLs detected for these traits overlapped on chromosomes I, V, and VIII. Our results should provide a basis for further studies on candidate genes affecting blackspot bruise susceptibility and enzymatic discoloration.

**Electronic supplementary material:**

The online version of this article (doi:10.1007/s00438-017-1387-0) contains supplementary material, which is available to authorized users.

## Introduction

Enzymatic discoloration (ED) and blackspot bruising affect potato tuber quality via the formation of dark pigments from phenolic compounds and the action of polyphenol oxidase (PPO) (Cobb 1999). The ED that appears after peeling and cutting the tuber leads to not only undesirable colours but also flavour changes and loss of nutritional quality (Vitti et al. 2011). ED occurs not only in potato tubers but also in fruits, vegetables, and seafood. Blackspot bruising of potatoes is an internal defect of tubers caused by mechanical impact and is observed only after peeling of the tuber skin and short exposure to the air. Bruising leads to rejection of the crop by consumers and the processing industry, thus resulting in considerable economic losses (Storey 2007). Potato bruising is estimated to cost the U.S. potato industry at least $298 million each year, as deduced from a total potato production worth $2 billion (Thorton and Bohl 1995). The British Potato Marketing Board has estimated losses caused by the potato damage to be £30 million per year, corresponding to approximately £200 per hectare (Anon. 1994). To decrease losses due to mechanical damages formed during harvest and at all stages of postharvest handling, the breeding of new potato cultivars with enhanced resistance to mechanical damage and blackspot bruising is an appropriate approach. The primary difficulty in the phenotypic selection of cultivars resistant to bruising in potato breeding programs is the limited number of tubers at an early stage of the breeding cycle. Potato breeding starts with a cross from which each true seed gives rise to a clonal, tuber-propagated line. The rate of vegetative propagation of such line is rather slow: eightfold per season (Bradshaw and Mackay 1994), while the methods of scoring tuber bruising are destructive for the assessed tubers. Diagnostic molecular tools for the trait would provide an advantage for breeding programs aimed at selection of cultivars resistant for bruising.

Blackspot bruising of potato tubers is a complex trait resulting from the interactions of both genetic and environmental factors (McGarry et al. 1996), with heritability values varying from 0.45 to 0.93 (Zgórska 1989; Pavek et al. 1993; Hara-Skrzypiec and Jakuczun 2013; Urbany et al. 2011a). The biochemical components of the blackspot bruise reaction, PPO, and its substrates are expected to be essential for melanin synthesis in both ED and blackspot bruising. The importance of PPO in the ED process has been confirmed by the absence of discoloration in tubers with silenced expression of the PPO gene (Bachem et al. 1994; Coetzer et al. 2001). Commercial companies have applied the PPO-silencing approach to create non-browning potatoes and apples (Walz 2015). Moreover, the low rate of discoloration in the wild species *Solanum hjertingii* is due to low PPO activity (Brown et al. 1999). Culley et al. (2002) have demonstrated that *S. hjertingii* has a truncated version of *POT32* (one of the isoforms of polyphenol oxidase), which is a major form of *PPO* expressed in tubers (Thygesen et al. 1995). Werij et al. (2007) have dissected the genetic factors responsible for the variation observed in ED using a diploid potato family as a mapping population. The authors have identified three QTLs for ED on potato chromosomes I, III, and VIII, and the QTL on chromosome VIII co-localizes with molecular markers for *POT32*. The authors have discriminated three different alleles of the *POT32* gene (*POT32PS1, POT32PS2*, and *POT32PS3*), and genotypes with two copies of allele *POT32PS1* have the highest levels of both *POT32* gene expression and discoloration. The complexity of the genetic control of bruising and the various PPO alleles segregating in the studied genetic material may be primary reasons for the weak correlation between the trait and PPO activity demonstrated in many studies (Stark et al. 1985; Stevens and Davelaar 1997; Lærke et al. 2000).

Tyrosine, a primary substrate of the discoloration reaction, is expected to be an important factor in bruising. The previous studies have demonstrated that the tyrosine level is correlated with bruising, as assessed as discoloration after tissue homogenization and in abrasive peeling tests (Corsini et al. 1992; Dean et al. 1993), although the results of these tests are more correlated with the level of ED than to level of bruising. However, weak or non-significant correlations between blackspot formation and tyrosine levels have been reported in studies using the impact method for bruising evaluation (Stevens and Davelaar 1997; Strehmel et al. 2010; Hara-Skrzypiec and Jakuczun 2013). The content of chlorogenic and caffeic acids, potential monophenolic substrates of PPO in tubers, is not correlated with ED levels and blackspot formation (Friedman 1997; Lærke et al. 2002a). In addition, as demonstrated by Werij et al. (2007), QTLs for chlorogenic acid and tyrosine content do not overlap with the QTLs for ED.

In addition to the biochemical aspects of blackspot bruise formation, structural factors, such as cellular stability, cell number and volume, cell wall thickness, and tuber turgidity, also influence tuber bruising (Aeppli et al. 1981; McGarry et al. 1996; Lærke et al. 2002b).

A close relationship between bruising susceptibility and tuber-specific gravity has been described in many reports (Scudder et al. 1950; Massey et al. 1952; Ophuis et al. 1958; Rogers-Lewis 1980; Corsini et al. 1999; Urbany et al. 2011a). However, most of these studies have also demonstrated that some of the tested cultivars do not follow this tendency. An explanation for the correlation between bruising and specific gravity/dry matter content may be the interaction of starch grains with cell membranes; cells full of starch grains are more vulnerable to impact damage, because starch granules may rupture the membranes during the impact (Hughes 1980).

Urbany et al. (2011a) have reported the first attempts to dissect the genetic factors underlying bruising susceptibility and have associated DNA polymorphisms at multiple functional candidate loci with phenotypic variations in tetraploid cultivars and breeding clones. This strategy has led to the identification of significant associations among markers for *PPO* isoforms, *hydroxycinnamoyl CoA quinate transferase (HCT), starch phosphorylase L-type (PHO1A)*, and *4-coumarate CoA ligase (4CL)* genes and tuber bruising susceptibility. By comparing the tuber proteomes of bruising-resistant and susceptible cultivars, Urbany et al. (2011b) have discovered a novel candidate gene for bruising: *triacylglycerol lipase III (LipIII27)*. Markers derived from this gene are significantly associated with bruising susceptibility.

The goal of our study was to perform mapping of QTLs for blackspot bruise susceptibility in diploid potatoes. This study is the first to use a full-sib linkage mapping and QTL analyses of the bruising susceptibility and enzymatic discoloration susceptibility. Our approach, based on a dense genetic map constructed of DArT markers, complements the previous approaches by including new data on bruising susceptibility inheritance. Two different test methods were applied to determine bruising susceptibility. To clarify relationships between the bruising susceptibility and tuber starch content observed in breeding practice, QTL analysis of tuber starch content-corrected bruising susceptibility (SCB_RD,_ SCB_FB_) was conducted and compared with the results of independent mapping the QTLs for bruising susceptibility and tuber starch content.

## Materials and methods

### Plant materials

Diploid potato clones DG 06-5 (susceptible to bruising) and DG 03-226 (resistant to bruising) were crossed to obtain the *F*_1_ mapping population 11–36 (*N* = 149). The parental forms resulted from a long-term recombinant breeding program at Plant Breeding and Acclimatization Institute—National Research Institute and were complex interspecific *Solanum* hybrids with *S. tuberosum, S. acaule, S. chacoense, S. demissum, S. gourlayi, S. microdontum, S. phureja, S. verrucosum*, and *S. yungasense* in their pedigree. In addition, DG 03-226 had a contribution from *S. stenotomum*. The theoretical contributions of *S. tuberosum* in the forms DG 06-5 and DG 03-226 were 70 and 69%, respectively. The corresponding values were 11 and 5% for *S. chacoense* and 6 and 14% for *S. phureja*. The mapping population, its parental forms and standard cultivars, table cv. Vineta and starch cv. Hinga, were planted at the end of April and harvested at the end of September in 3 consecutive years (2012, 2013, and 2014). The field experiments were fertilized with 90, 90, and 170 kg ha^−1^ of N, P, and K, respectively, and potatoes were chemically protected against pests and pathogens.

### Phenotypic and statistical analyses

Blackspot bruise susceptibility was determined with two methods. In the first method, blackspot bruises were induced in a rotating drum (*B*_RD_) according to Domański et al. (2007). Experiments were conducted in mid-February in 2013, 2014, and 2015 with two (2013, 2015) or three replicates (2014). To assess the resistance to blackspot bruising, each replicate included 14–20 tubers per genotype. The selected tubers were undamaged, non-greening, and, as much as possible, uniform in size within each genotype. Sample tubers were stored for 5 months at 5–10 °C to simulate a storage period common in the potato industry. Before the test, tubers were incubated for 12 h at 11 °C. Tubers were placed in a hexagonal drum that was rotated ten times to stimulate the formation of blackspots (Douches et al. 2003). Then, the bruised tubers were stored at 20 °C for 72 h. After peeling, blackspot bruises were visually evaluated and then divided into four quality groups: *A* = 0–25% of tuber surface covered by bruises, lack of or weak bruising; *B* = 25–50% of tuber surface covered by bruises, moderate bruising; *C* = 50–75% of tuber surface covered by bruises, strong bruising; and *D* = 75–100% of tuber surface covered by bruises, very strong bruising. A bruising index was calculated with the formula *B*_RD_ = [(0.25*A* + 0.5*B* + 0.75*C* + *D*)/number of tested tubers] × 100. The *B*_RD_ ranged from 0 (resistant to blackspot bruising) to 100 (the most susceptible to bruising) (a modified method according to Urbany et al. 2011a). The second method, the falling bolt method (*B*_FB_), was modified as described by Johnson et al. (2003). Experiments were conducted in mid-February in 2014 and 2015. The evaluations of *B*_FB_ were performed on two (2015) or three replicates (2014). Incubation conditions before and after impact were the same as those in the *B*_RD_ method. Five tubers from each genotype were tested per replicate. The tubers were subjected to mechanical impact involving a metal bolt imparting 0.76 J of energy. After incubation at 20 °C for 72 h, each tuber was transected through the site of mechanical impact. The spot sizes (diameter and depth in mm) and colour intensity of the bruises were rated according to the scale of Hironaka et al. (1996): 0 = no discoloration; 1 = very small spots or vaguely defined; 2 = spot diameter 3–5 mm, depth less than 5 mm, grey or brownish; 3 = spot diameter 5–10 mm, depth less than 5 mm, grey or black; 4 = spot diameter 5–10 mm, depth greater than 5 mm, grey or black; and 5 = spot diameter greater than 10 mm, depth greater than 5 mm, black.

Tuber starch content (TSC, %) was estimated with the underwater weight method, on the basis of the ratio of tuber weight in air to tuber weight in water, according to Lunden (1956).

Starch-corrected blackspot bruise susceptibility estimated by the rotating drum (SCB_RD_) and falling bolt (SCB_FB_) methods was calculated using regression analysis according to Urbany et al. (2011a).

Enzymatic discoloration was evaluated in December 2012, 2013, and 2014. Five tubers from each genotype were tested in two (2012, 2014) or three replicates (2013). The degree of discoloration was evaluated 4 h after the tuber was cut in half. The level of ED was scored according to the colour chart for determining the discoloration of potato (Dansk Gærings-Industri, Ltd., Copenhagen, Denmark) on a scale of 1–9 (where 1 = the strongest discoloration, and 9 = lack of discoloration).

The normality of the distribution of phenotypic data was tested with the Shapiro–Wilk test. The reproducibility of analysed traits between years was estimated by calculating linear Pearson’s correlation coefficients. The determination coefficients (*R* ^2^) for blackspot bruising were estimated from an analysis of variance. The broad-sense heritability was estimated according Domański et al. (2007) according to the formula: *H*_b_ = *σ* ^2^_g_/*σ* ^2^_g_ + *σ* ^2^_ge_ + *σ* ^2^_e_. All statistical analyses were performed using STATISTICA for Windows (StatSoft Polska, Kraków, Polska).

### Genetic mapping and QTL analysis

Genomic DNA was isolated from freeze-dried leaf tissue using a GeneElute Plant Genomic DNA Miniprep kit (Sigma-Aldrich, St. Louis, Missouri, USA). Markers derived from the candidate genes listed in Supplementary Table S1 were amplified in 20 μl of PCR mixture containing 2 μl of 10 × PCR buffer (Sigma-Aldrich, St. Louis, Missouri, USA), 6 µM MgCl_2_ (25 mM), 100 µM dNTPs (Sigma-Aldrich, St. Louis, Missouri, USA), 0.4 µM each primer, 1 U DreamTaq polymerase (Fermentas Life Sciences, Thermo Fischer Scientific, Inc.), and 10–30 ng of the template DNA. The PCR parameters were as follows: 94 °C for 60 s and 40 cycles of 93 °C for 25 s, 50–65 °C for 35 s, 72 °C for 90 s, and 72 °C for 300 s (Supplementary Table S1). Restriction digestions were performed in 20 µl of reaction mixture containing 10 µl of PCR product, 1 µl of enzyme (listed in Table S1), 2 µl of buffer provided by the enzyme supplier, and 7 µl of H_2_O for 3 h at the temperature recommended by the enzyme supplier (37 or 65 °C). DNA polymorphisms in amplicons with or without restriction endonuclease digestion site were detected by electrophoresis in 1.5% agarose gels with ethidium bromide staining. Diversity Array Technology (DArT) analysis was performed by Diversity Array Pty Ltd., Canberra, Australia, as described in Śliwka et al. (2012a, b), using protocols developed for other plant species (Jaccoud et al. 2001; Wenzl et al. 2004; Akbari et al. 2006). Parental linkage maps and a common map were constructed using the JoinMap^®^4 software with the following settings: CP (cross pollinator) population type, independence LOD (logarithm of odds) as a grouping parameter (linkages with LOD > 3 were considered significant), regression mapping algorithm, and Haldane’s mapping function (Śliwka et al. 2012a). Identification and orientation of particular potato chromosomes were performed by comparing the maps with those from the previous DArT mapping studies in *Solanum* (Śliwka et al. 2012a, b; Sharma et al. 2013). Separate linkage maps for both parents were constructed using the function Create Maternal and Paternal Population Nodes, which analysed only markers descending from the given parent, whereas the ones descending from the other parent or from both parents were ignored. Then, a common map based on all markers was constructed using the function Create Population Node. QTL analysis was performed using interval mapping with the MapQTL^®^6 software (Van Ooijen 2009). QTLs were detected using an LOD threshold ≥ 3.1.

## Results

### Phenotyping evaluation

The mean values of *B*_RD_, *B*_FB_, SCB_RD_, SCB_FB_, ED and TSC of the parents and individuals from the mapping population 11–36 and standard cultivars are presented in Table 1. The paternal clone DG 03-226 was highly resistant to bruising and enzymatic discoloration with a low value of TSC. The maternal clone DG 06-5 was highly susceptible to bruising with high TSC.

**Table 1:** Mean values of *B*_RD_, *B*_FB_, SCB_RD_, SCB_FB_, ED and TSC (%) of the parents and individuals from the mapping population 11–36 and standard cultivars, ranges of the traits and standard deviations

Trait^a^	DG 03-226 ♂	DG 06-5 ♀	Standard cv.Vineta	Standard cv.Hinga	*F*_1_, *N* = 149 population mean	Range of *F*_1_ variation
*B*_RD_	9.3 (± 1.9)	87.3 (± 0.4)	1.2 (± 0.9)	61.7 (± 8.3)	46.9 (± 17.6)	5.0–91.1
*B*_FB_	1.4 (± 0.3)	4.8 (± 0.3)	0.0 (± 0.0)	1.9 (± 1.0)	2.0 (± 1.0)	0.1–4.5
SCB_RD_	− 34.9 (± 1.2)	29.5 (± 4.1)	− 35.5 (± 1.1)	9.9 (± 5.6)	− 1.1 (± 16.3)	− 40.5–39.9
SCB_FB_	− 0.8 (± 0.2)	2.1 (± 0.1)	− 1.4 (± 0.02)	− 0.5 (± 0.04)	− 0.008 (± 0.9)	− 1.9–2.9
ED	8.2 (± 0.4)	5.3 (± 0.5)	8.5 (± 0.5)	6.7 (± 1.4)	7.4 (± 0.9)	5.3–9.0
TSC	16.4 (± 1.0)	24.8 (± 2.7)	12.4 (± 0.7)	21.1 (± 2.8)	18.7 (± 2.3)	11.6–23.4

^a^Mean values are calculated from all years and replications; *N* number of analysed *F*_1_ individuals

Pearson’s *r* values for *B*_RD_ between results from specific years ranged from 0.65 to 0.77 and for SCB_RD_ from 0.63 to 0.75. Pearson’s correlation coefficient for *B*_FB_ between tested years was *r* = 0.56 and for SCB_FB_ *r* = 0.52. Pearson’s correlation coefficients for ED and TSC between tested years ranged from 0.54 to 0.72 and from 0.78 to 0.81, respectively. Pearson’s correlation coefficients between tested years for all traits were significant at *α* = 0.0001.

Values of *B*_RD_, *B*_FD,_ SCB_RD_, SCB_FD_, and TSC were normally distributed in the mapping population 11–36, whereas ED values deviated significantly from normality, with a distribution skewed towards low discoloration (Supplementary Fig. S1).

Analysis of variance in the mapping population demonstrated significant effects of genotype, year, and interaction genotype × year on *B*_RD_, *B*_FB_, SCB_RD_, SCB_FD,_ ED, and TSC (Table 2). Genotype had the largest influence on all six assessed traits, explaining from 53.2% for SCB_FB_ up to 63.5% of the variance for B_RD_. For four of six traits, the effects of the genotype × year interactions were stronger than the year effects, which was not the case for SCB_RD_ and TSC.

**Table 2:** Analysis of variance in *B*_RD_, *B*_FB_, SCB_RD_, SCB_FB_, ED and TSC in the mapping population 11–36

Factor/interaction	*Df* effect^a^		Mean sum of squares effect		*Df* error^a^	Mean sum of squares error		*F*	*P*	*R* ^2^ (%)^b^
*B*_RD_										
Genotype {1}	148.0		2037.0		543.0	102.3		19.9	0.00000	63.5
Year {2}	2.0		24231.0		543.0	102.3		236.9	0.00000	10.2
1 × 2	283.0		243.7		543.0	102.3		2.4	0.00000	14.5
*B*_FB_										
Genotype {1}	146.0		4.4		433.0	0.7		6.2	0.0000	56.8
Year {2}	1.0		9.1		433.0	0.7		12.8	0.0004	0.8
1 × 2	143.0		1.2		433.0	0.7		1.7	0.0000	15.4
SCB_RD_										
Genotype {1}	148.0		1767.6		543.0	95.4		18.5	0.00000	58.3
Year {2}	2.0		34725.5		543.0	95.4		364.1	0.00000	15.5
1 × 2	283.0		233.1		543.0	95.4		2.4	0.00000	14.7
SCB_FB_										
Genotype {1}	146.0		4.4		432.0	0.7		6.2	0.00000	53.2
Year {2}	1.0		9.0		432.0	0.7		12.6	0.000418	1.8
1 × 2	143.0		1.2		432.0	0.7		1.7	0.000015	16.2
ED										
Genotype {1}	148.0		5.5		585.0	0.5		10.1	0.00000	53.9
Year {2}	2.0		54.2		585.0	0.5		99.4	0.00000	7.2
1 × 2	289.0		0.9		585.0	0.5		1.7	0.00000	17.6
TSC										
Genotype {1}	148.0		35.6		588.0	1.6		22.1	0.00000	54.1
Year {2}	2.0		1369.6		588.0	1.6		849.9	0.00000	28.1
1 × 2	292.0		2.7		588.0	1.6		1.7	0.00000	8.0

^a^Number of degrees of freedom

^b^Percent of variance explained

The broad-sense heritabilities of blackspot bruise susceptibility evaluated via the rotating drum and falling bolt methods were *H*_b_ = 0.71 and *H*_b_ = 0.68, respectively. The broad-sense heritabilities of starch-corrected *B*_RD_ and starch-corrected *B*_FB_ were *H*_b_ = 0.67 and *H*_b_ = 0.57, respectively. The broad-sense heritabilities of ED and TSC were *H*_b_ = 0.62 and *H*_b_ = 0.80, respectively.

The evaluations of blackspot bruising susceptibility with the two methods were highly correlated (Table 3). The correlation analyses of the phenotypic data demonstrated that genotypes with higher TSC were more susceptible to blackspot bruise formation than those with low TSC. Genotypes with high susceptibility to bruising also demonstrated strong ED. However, the correlation between TSC and ED was not significant.

**Table 3:** Correlation coefficients (Pearson’s *r* values) *B*_RD_, *B*_FB_, SCB_RD_, SCB_FB_, ED and TSC in mapping population 11–36

	*B*_RD_	*B*_FB_	SCB_RD_	SCB_FB_	ED
*B*_FB_	0.70**				
SCB_RD_	0.97**	0.67**			
SCB_FB_	0.64***	0.96**	0.66**		
ED	0.51^a^***	0.63^a^***	0.53^a^***	0.64^a^***	
TSC	0.42***	0.32***	0.21*	n.s	n.s

*n.s*. not significant

^a^Scale for ED evaluation was reversed (9 = the strongest discoloration) for the purpose of correlation coefficients calculations

^*^Significant at *α* = 0.01

^**^Significant at *α* = 0.001

^***^Significant at *α* = 0.0001

### Linkage map

**Fig. 1:**
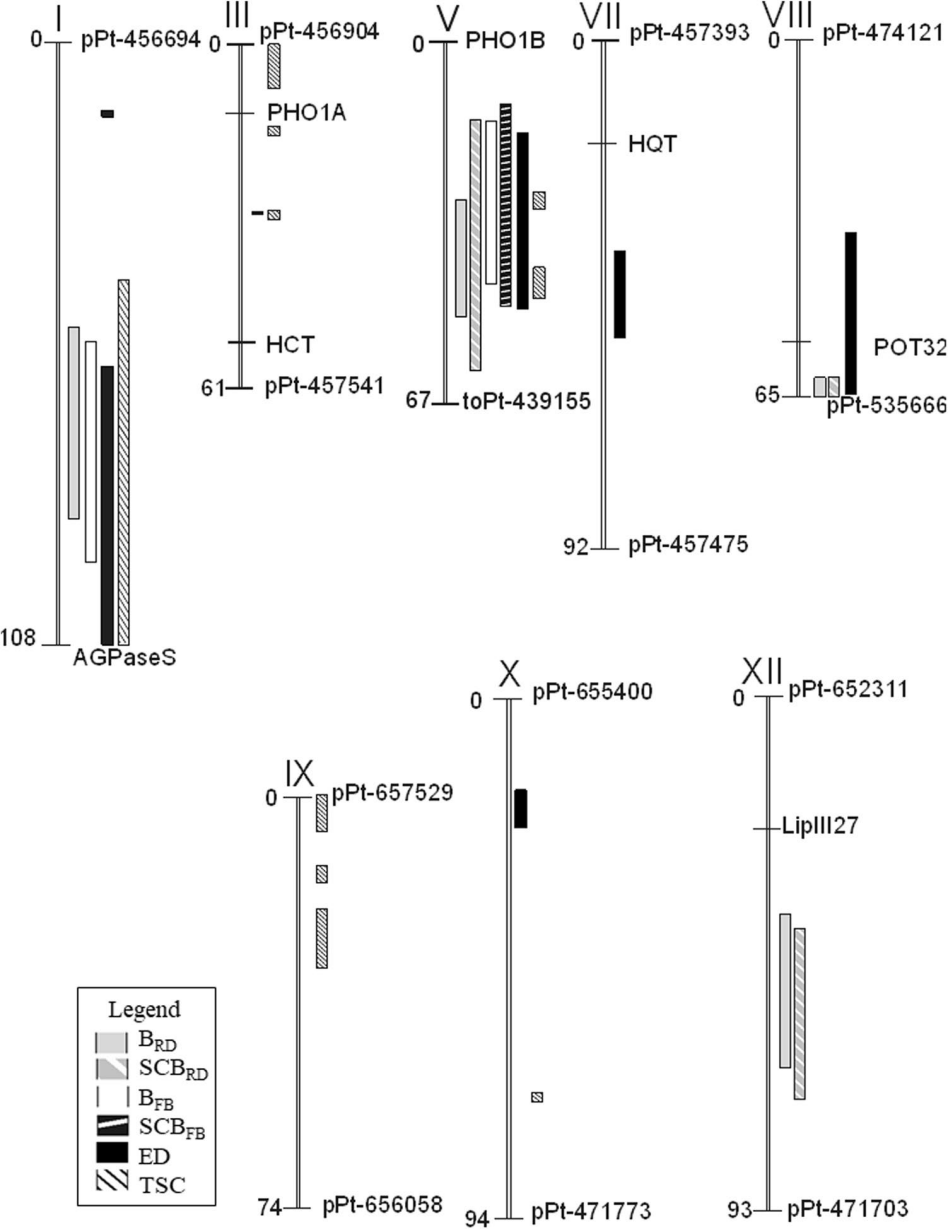
QTL map of population 11–36 for *B*_RD_, SCB_RD_, *B*_FB_, SCB_FB_, ED and TSC. In addition, map position of the candidate genes is shown

Of the 5363 DArT markers scored in mapping population 11–36, 2564 DArT markers segregated. After exclusion of markers with more than 10% missing data (192 markers), those with missing parental data (91 markers), and those with quality parameters below thresholds of *p* < 60 (288 markers) and call rate < 85 (112 markers), 1881 DArT markers were used to construct a genetic map. Markers with identical patterns of segregation were excluded with the JoinMap®4 program. An initial grouping in the population node (markers from both parents analysed simultaneously) yielded 19 groups with the average LOD for grouping 10.7 (range 9–15). On the basis of the strongest cross-link (SCL) parameter and the presence of markers with known chromosomal positions, 12 groups corresponding to potato chromosomes were formed. The LOD for mapping varied from 13.6 to 42.8 in particular groups, with the average LOD 25.6. The final genetic map common for parents consisted of 1359 DArT markers and nine sequence-characterized amplified region (SCAR) and cleavage-amplified polymorphic sequence (CAPS) markers. Of these 1368 markers, 390 originated from parent DG 03-226, 452 originated from parent DG 06-5, and 526 originated from both parents. The total length of the genetic map reached 972 cM. The lengths of the chromosomes ranged from 54 (chromosome IV) to 109 cM (chromosome I). The numbers of markers located on particular chromosomes varied from 48 on chromosome IV to 167 on chromosome II (Fig. 1, Supplementary Table S2).

### QTL analysis

QTLs detected using the mean data sets are shown in Table 4, whereas QTLs detected in data sets from particular years of phenotyping are presented in supplementary table S3. QTLs significant only in some years appear only in supplementary table S3.

**Table 4:** QTL detected for mean *B*_RD_, *B*_FB_, SCB_RD_, SCB_FB_, ED, TSC in the mapping population 11–36. Only QTL with LOD ≥ 3.1 are presented

Chromosome	Trait	Marker at peak or markers flanking virtual interval	Marker origin^a^	Peak position (cM)	LOD	*R* ^*2*^(%)	Interval (cM)
I	*B*_RD_	toPt-437458	H	75.4	5.00	14.3	51.5–86.8
	*B*_FB_	pPt-538190	P2	72.9	6.76	19.1	54.1–94.8
	ED	pPt-538519	H	13.7	3.17	9.3	13.6–14.7
		toPt-437458	H	75.4	6.66	18.3	57.4–108.8
	TSC	pPt-538418	H	75.7	8.49	23.1	43.1–108.8
III	TSC	pPt-456904	H	0.0	4.85	13.9	0.0–7.9
		pPt-472721- pPt-536347	H	14.1	3.56	10.4	13.0–15.0
		pPt-458741	P2	32.4	3.84	11.2	25.4–32.4
	ED	pPt-473371- pPt-540060	H	29.7	3.96	11.5	28.7–30.7
V	*B*_RD_	pPt-539905	H	24.5	4.94	14.2	22.2–45.1
	*B*_FB_	pPt-539905	H	24.5	5.41	15.6	13.6–43.2
	SCB_RD_	pPt-655798	P2	31.6	7.77	21.4	13.6–60.1
	SCB_FB_	pPt-540393	H	32.5	8.92	24.4	11.6–47.6
	ED	pPt-650647	H	26.0	7.57	20.9	11.6–44.1
	TSC	pPt-652324	P1	25.3	3.48	10.2	25.3–25.4
		pPt-559550	H	40.7	3.81	11.1	35.9–41.6
VII	ED	pPt-654764- pPt-653593	H,P2	47.3	3.78	11.0	37.4–54.3
VIII	*B*_RD_	pPt-535666	P1	65.2	3.24	9.5	61.7–65.2
	SCB_RD_	pPt-535666	P1	65.2	3.11	9.2	61.7–65.2
	ED	pPt-535666	P1	65.2	4.84	13.9	35.3–65.2
IX	TSC	pPt-657529- pPt-656452	H,P2	1.0	4.54	13.1	0.0–5.5
		pPt-473072	H	13.5	4.62	13.3	11.3–15.7
		pPt-471870	H	33.6	3.77	11.0	29.6–34.7
X	ED	pPt-456962	P2	15.6	4.00	11.6	15.6–23.7
	TSC	pPt-652232	H	72.2	3.43	10.1	71.7–72.2
XII	*B*_RD_	pPt-651718	H	49.2	4.88	14.0	42.0–69.6
	SCB_RD_	pPt-651718	H	49.2	4.81	13.8	43.7–72.6

The most significant QTL for each trait is marked in bold

*H* descending from both parents

^a^P1-inherited from DG 03-226. P2-inherited from DG 06-5

In mapping population 11–36, QTLs on chromosome V were significant for all six tested traits (mean data sets and most data sets from specific years, Table 4 and Supplementary Table S3, Fig. 1).

QTLs for mean *B*_RD_ were detected on chromosomes I, V, VIII, and XII (Table 4). The most significant QTL for *B*_RD_ was detected on chromosome I and was significant in three of four data sets, whereas the QTLs for *B*_RD_ on chromosomes V and XII were significant in all data sets.

The QTLs for *B*_FB_ were significant in all three data sets on chromosomes I and V. The QTL peaks for *B*_RD_ and *B*_FB_ on chromosome V were in the same position (24.5 cM).

QTLs for SCB_RD_ were detected on chromosomes V, VIII, and XII. The most significant QTL was on chromosome V, and the QTLs on chromosome XII were significant in all four data sets (SCB_RD_ and SCB_RD_12–14). QTL peaks for B_RD_ and SCB_RD_ on chromosomes VIII and XII were in the same positions, and the effects of these QTLs were similar. The QTL for SCB_FB_ was detected on chromosome V and was significant for all data sets.

QTLs for ED were detected on chromosomes I, III, V, VII, VIII, and X. Three QTLs for ED, which were significant in all four data sets (ED and ED12–ED14), were detected on chromosomes I, V and VIII. The most important QTL for ED was on chromosome V. The QTL localizations for *B*_RD_ and ED overlapped on chromosomes I and VIII. The QTL peaks for these traits were localized in the same positions.

The most important QTL for tuber starch content was detected on chromosome I, spanning a wide region of the chromosome, from 43.1 to 108.8 cM, with the peak at 75.7 cM. Another QTL for TSC, which was significant in all four data sets (TSC and TSC12-TSC14), was localized on chromosome IX. Two other QTLs for TSC also on chromosome IX were significant for mean TSC, TSC12, and TSC14. Three QTLs on chromosome III and the QTL on chromosome X were significant for mean TSC and 2 years of testing. Two QTLs on chromosome V were detected only in 1 year of testing.

### The candidate gene approach

The genetic positions of nine markers derived from candidate genes and their physical positions in the potato DM 1–3 516 R44 (DM1-3) genome (v4.03) (http://solanaceae.plantbiology.msu.edu/integrated_searches.shtml) are presented in supplementary table S1. In our study, *LipIII27* was mapped to chromosome XII, which is a location different from the published location on chromosome II of the sequence used to design *LipIII27*. Only two of nine tested sequence-specific markers were significant for the analysed traits. The marker AGPaseS mapped within the QTLs for ED and TSC on chromosome I. AGPaseS explained 12.7% (LOD = 4.40) of the variation for ED and 12% (LOD = 4.14) of the variation for TSC. The marker for the *POT32* gene, located on chromosome VIII, was significant for ED and explained 11.8% (LOD = 4.07) of the variation for this trait. None of the chosen markers were significantly related to blackspot bruising susceptibility evaluated with the two methods.

## Discussion

Both blackspot bruise susceptibility and ED depend on multiple factors. According to the analysis of variance in our study, genetic factors were the most important in their determination (Table 2). Simultaneously, the genotype × year interaction effects were larger than year effects for bruising susceptibility and enzymatic discoloration, but this trend was not found for starch content. Heritability in a broad sense was high for all tested traits but was the highest for TSC, followed by *B*_RD_, *B*_FB_, SCB_RD_, ED. and SCB_FB_.

Blackspot bruise susceptibility is a complex trait that cannot be ascribed to any single physiological parameter. The relationships among various physiological parameters and bruising susceptibility change depending on the impact method (Lærke et al. 2000). Hence, determination of the overall mechanism of blackspot bruise susceptibility in our study included evaluation of the trait with two impact methods, rotating drum and falling bolt, in combination with evaluation of discoloration potential after tuber cutting (ED). The applied impact procedures affect both the physical and the biochemical properties of tubers. While the falling bolt method provided precise and standardized information on the tissue reaction to the impact of controlled strength, the rotating drum method, which was meant to mimic packing plant conditions, resulted in scoring the final bruising extent as an effect of many factors including the tissue reaction, tuber size, and shape.

**Fig. 2:**
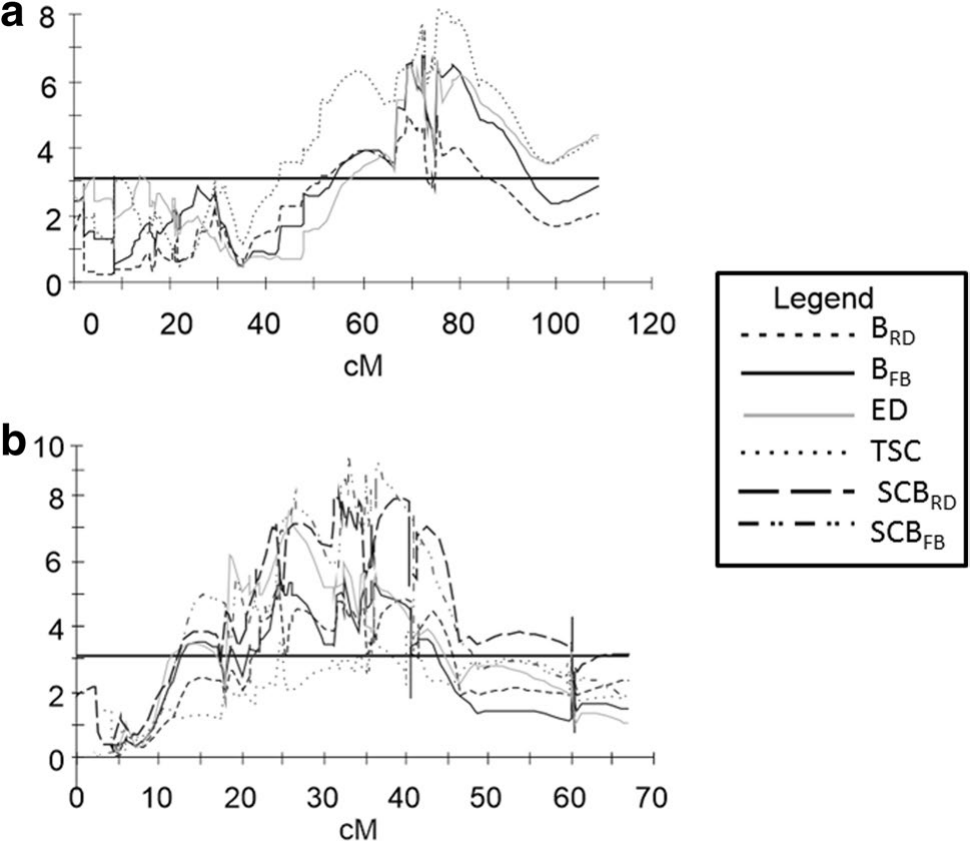
QTL on chromosome I (**a**) and chromosome V (**b**) for mean *B*_RD_, *B*_FB_, SCB_RD_, SCB_FB_, ED and TSC. The threshold LOD score (LOD = 3.1) is indicated by horizontal line

Despite the comparisons that indicate considerable differences among test methods (Aeppli et al. 1981; Lærke et al. 2002b), in our study, a highly significant correlation was detected between the falling bolt and the rotating drum methods. The most significant QTLs for *B*_RD_ and *B*_FB_ overlapped on chromosome I. Other important QTLs for *B*_RD_ and *B*_FD_ were detected for all data sets on chromosome V, with the same peak position (Fig. 2). However, the rotating drum method detected an additional QTL for the trait on chromosome XII, which was significant in all tested years. The most important QTLs for blackspot bruise susceptibility were significant in three or four data sets, thus indicating that the trait was relatively stable across different vegetation seasons.

To clarify relationships between bruising susceptibility and tuber starch content, observed in breeding practice and confirmed in correlation analysis in our study, QTL mapping of starch content-corrected bruising susceptibility was conducted. The major effect QTL for bruising detected on chromosome I overlapped with the most significant QTL for tuber starch content and was not detected for starch-corrected bruising. The most significant QTLs for SCB_RD_ and SCB_FB_ were detected on chromosome V, and their effects were enhanced in comparison with those of the QTLs for *B*_RD_ and *B*_FB_. Partial overlap of QTLs for SCB_RD_ and SCB_FB_ and for TSC on chromosome V may result from linked genes that control both traits independently. The major QTL for bruising not related to starch content on chromosome V, and the two weaker QTLs on chromosomes VIII and XII provide information on the genetics of bruising susceptibility, rather than providing information to guide the selection of resistant clones independently of their starch content.

In our study, the QTL analysis of ED revealed the contribution of seven QTLs to the trait. These QTLs were located on six chromosomes: I, III, V, VII, VIII, and X. The QTL on chromosome VIII co-localized with the marker for *POT32*, thus confirming the results of Werij et al. (2007). Werij et al., in their first report of genetic factors underlying ED, detected QTLs for ED on chromosomes VIII (explaining 21% of total variance), I, and III. In our study, the major QTL for ED (explaining 20.9% of variance) was localized on chromosome V and co-localized with QTLs for *B*_RD_, *B*_FB,_ SCB_RD_, SCB_FB_, and TSC. The QTLs on chromosomes VII and X were specific for the trait and did not co-localize with QTLs for other analysed traits. The region on chromosome V, together with the QTL on chromosome VIII, is apparently essential for bruising and enzymatic discoloration formation, and may include genes affecting pigment synthesis both after bruising and after tissue cutting.

Possible candidate genes located on the DM1-3 physical map within a region corresponding to the QTLs for *B*_RD_, *B*_FB_, SCB_RD_, SCB_FB_, and ED on chromosome V on genetic map 11–36 were *phenylalanine ammonia-lyase* (PGSC000DMG400005492; chr05:36342746..36347409) and *peroxidase* (PGSC0003DMG400005279; chr05:42523943..42525912). The two genes encode proteins involved in the production and the oxidative reactions of phenolic compounds, respectively (Supplementary Table S4). However, the QTL region spans a large portion of the chromosome V with many other putative candidates including other peroxidases as well.

We identified ten QTLs for starch content on five chromosomes: I, III, V, IX, and X. The most important QTL for TSC was detected on chromosome I, spanning a wide region of the chromosome, from 43.1 to 108.8 cM. The effect of this QTL on the mean TSC explained 23.1% of the variance. The marker AGPaseS was located within this QTL and explained 12% of the TSC variation. *AGPaseS* is one of the three expressed genes encoding the large subunit of the *AGPaseS* gene, which converts G1P and ATP into PPi and ADP-glucose (Sonnewald and Kossmann 2013). The location of *AGPaseS* has previously been reported on chromosome I and was associated with tuber starch content and starch yield (Chen et al. 2001). Some allele-specific sequences of this gene have been characterized in tetraploid potatoes and used as diagnostic markers for TSC (Li et al. 2013). The *AGPaseS* gene has also been mapped by Śliwka and co-workers (2016) in a diploid mapping population in the major QTL for TSC on chromosome I. Several authors have detected similar QTLs for TSC/specific gravity, as in our study, on chromosome V in diploid (Freyre and Douches 1994; Werij et al. 2012; Schäfer-Pregl et al. 1998; Manrique-Carpintero et al. 2015) and tetraploid potatoes (Bradshaw et al. 2008; McCord et al. 2011). Schäfer-Pregl et al. (1998), Werij et al. (2012) and Manrique-Carpintero et al. (2015) have also detected QTLs for TSC in positions similar to those observed in this study on chromosome IX.

None of the nine tested sequence-specific markers derived from genes chosen on the basis of published results (Werij et al. 2007; Urbany et al. 2011a, b; Śliwka 2016) were significant for blackspot bruise susceptibility in our study. Urbany et al. (2011a, b) have combined a proteomics approach using genotype groups with high and low bruising susceptibility with association mapping. With this strategy, the coincidence between differentially expressed proteins and bruising-associated DNA-based markers specific for protein-coding genes was tested. The authors have suggest that, among others, *polyphenol oxidase* isoforms *POT32* and *PHO1A* are strong candidate genes for susceptibility to bruising. The lack of linkage in our study between QTLs for bruising susceptibility and the candidate genes identified by Urbany et al. (2011a, b) might have resulted from use of different materials. The diploid parental forms used in our study were interspecific *Solanum* hybrids with several *Solanum* species in their pedigrees. The occurrence of gene alleles in diploid parents that are different from the alleles detected in tetraploid potato genotypes used by Urbany et al. (2011a, b) may be a reason for the identification of different genomic regions that were important for bruising susceptibility.

Marker LipIII27, derived from *triacylglycerol lipase III* (SGN-U269327), was mapped in our study to chromosome XII, which was a different location from that of the published sequence on chromosome II. Urbany et al. (2011b), through comparison of the tuber proteomes of bruising-resistant and susceptible cultivars, have identified this gene as a candidate gene for bruising susceptibility. In our study, marker LipIII27 did not co-segregate with the QTL for *B*_*R*D_ on chromosome XII. Another gene encoding triacylglycerol lipase III (PGSC0003DMG400028858; chr12:53245891..53249383) was located within the DM1-3 region corresponding to the QTL for *B*_RD_ (chr12:7849989..7850206—Unknown) and might contribute to its effect.

Novel regions within the potato genome influencing bruising not related to starch content were identified on chromosomes V, VIII, and XII. They represent a substantial contribution to the understanding this complex and important potato quality trait. Our work also provides new information about regions on chromosomes V, VII, and X not previously identified as important for enzymatic discoloration formation. The results may provide a basis for further investigations to identify new candidate genes responsible for the analysed traits.

## Electronic supplementary material

Below is the link to the electronic supplementary material.

**Fig. S1**. Distribution of blackspot bruise susceptibility estimated by rotating drum (B_RD_, in scale from 0—resistant to 100—the most susceptible to bruising), blackspot bruise susceptibility estimated by falling bolt (B_FB,_ in scale 1-5, where 1 = resistant to bruising, 5 = susceptible to bruising), starch-corrected blackspot bruise susceptibility estimated by rotating drum (SCB_RD_, obtained from B_RD_ and TSC according Material and Methods), starch-corrected blackspot bruise susceptibility estimated by falling bolt (SCB_FB_, obtained from B_FB_ and TSC according Material and Methods), enzymatic discoloration (ED, in scale 1-9, where 1 = the strongest discoloration, 9 = lack of discoloration), and tuber starch content (TSC, % FW) in population 11–36. Histograms and the normal distribution curves were generated using STATISTICA for Windows (Stat Soft, Inc. and StatSoft Polska Ltd., Polska). The normality of distribution of phenotypic data was tested by the Shapiro–Wilk test. ♂- DG 03-226, ♀ DG06-5. (TIF 193 KB)

**Fig. S2**. Number of markers and length of chromosomes (cM) of genetic map constructed for population 11-36 (N = 149) (JoinMap^®^4. Van Ooijen 2006) (TIF 39 KB)

Supplementary material 3 (XLSX 13 KB)

Supplementary material 4 (XLSX 53 KB)

Supplementary material 5 (DOCX 20 KB)

Supplementary material 6 (DOCX 16 KB)
