# Supplementary figures and images for: Quantitative trait loci for tuber blackspot bruise and enzymatic discoloration susceptibility in diploid potato
Source: Mol Genet Genomics. 2017 Oct 27;293(2):331–42. doi: 10.1007/s00438-017-1387-0 (PMC5854731; doi:10.1007/s00438-017-1387-0)

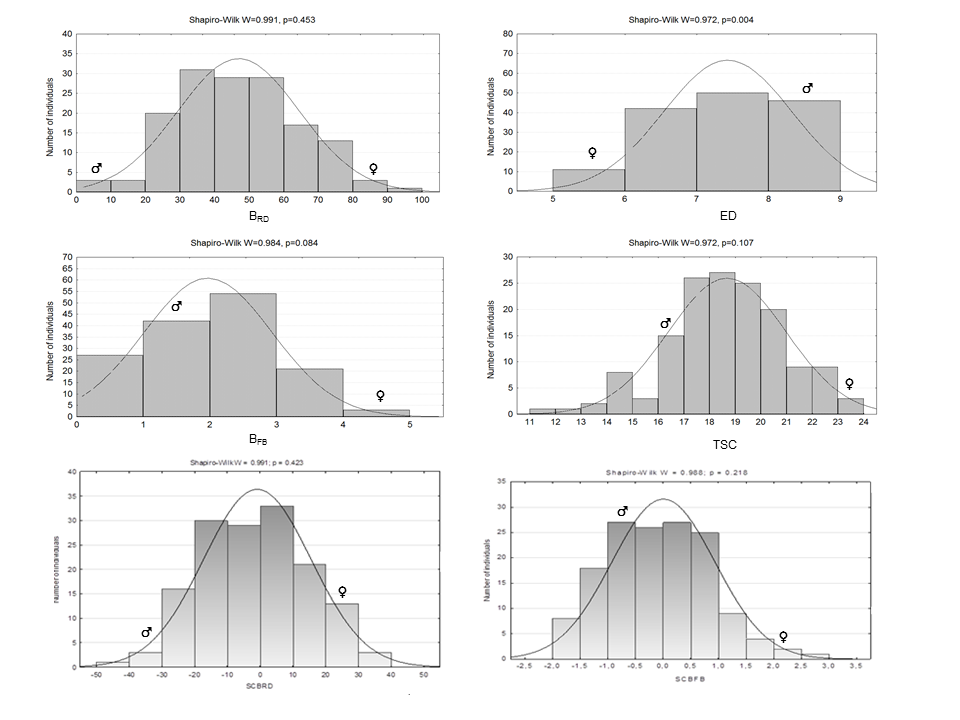

Supplement: Supplementary file 1 — Fig. S1. Distribution of blackspot bruise susceptibility estimated by rotating drum (BRD, in scale from 0—resistant to 100—the most susceptible to bruising), blackspot bruise susceptibility estimated by falling bolt (BFB, in scale 1-5, where 1 = resistant to bruising, 5 = susceptible to bruising), starch-corrected blackspot bruise susceptibility estimated by rotating drum (SCBRD, obtained from BRD and TSC according Material and Methods), starch-corrected blackspot bruise susceptibility estimated by falling bolt (SCBFB, obtained from BFB and TSC according Material and Methods), enzymatic discoloration (ED, in scale 1-9, where 1 = the strongest discoloration, 9 = lack of discoloration), and tuber starch content (TSC, % FW) in population 11–36. Histograms and the normal distribution curves were generated using STATISTICA for Windows (Stat Soft, Inc. and StatSoft Polska Ltd., Polska). The normality of distribution of phenotypic data was tested by the Shapiro–Wilk test. ♂- DG 03-226, ♀ DG06-5. (TIF 193 KB) [file 438_2017_1387_MOESM1_ESM.tif]

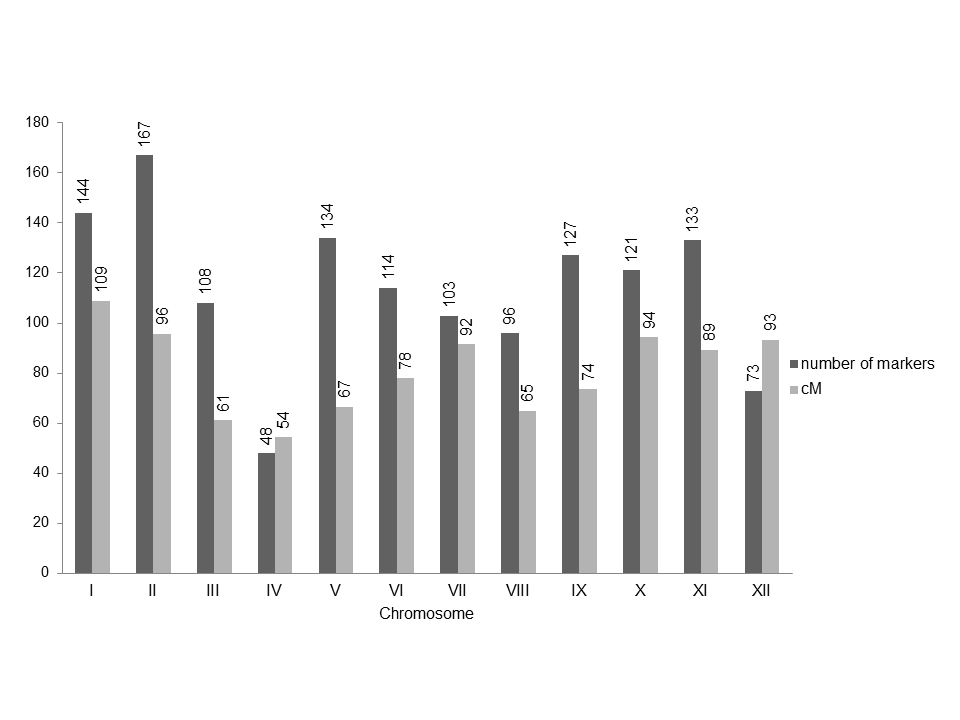

Supplement: Supplementary file 2 — Fig. S2. Number of markers and length of chromosomes (cM) of genetic map constructed for population 11-36 (N = 149) (JoinMap®4. Van Ooijen 2006) (TIF 39 KB) [file 438_2017_1387_MOESM2_ESM.tif]
