# Supplementary material for: Quantitative trait loci for tuber blackspot bruise and enzymatic discoloration susceptibility in diploid potato
Source: Mol Genet Genomics. 2017 Oct 27;293(2):331–42. doi: 10.1007/s00438-017-1387-0 (PMC5854731; doi:10.1007/s00438-017-1387-0)
Supplement: Supplementary file 5 — Supplementary material 5 (DOCX 20 KB) [file 438_2017_1387_MOESM5_ESM.docx]

Quantitative trait loci for tuber blackspot bruise and enzymatic discoloration susceptibility in diploid potato.

A. Hara-Skrzypiec, J. Śliwka, H. Jakuczun, E. Zimnoch-Guzowska

Plant Breeding and Acclimatization Institute – National Research Institute, Młochów, Platanowa 19, 05-831Młochów, Poland.

*Corresponding author: Agnieszka Hara-Skrzypiec; a.hara@ihar.edu.pl

Supplementary Table S3 QTL detected in specific years of phenotyping in the mapping population 11-36. Only QTL with LOD≥3.1 are presented.

| Chromosome | Trait | Marker/ interval | Marker origin^a/^ | Peak position (cM) | LOD | *R^2^*(%) |
| --- | --- | --- | --- | --- | --- | --- |
| I | B_RD_13 | toPt-437458 | H | 75.4 | 4.14 | 12.6 |
|  | B_RD_14 |  |  |  | 4.91 | 14.5 |
| V | B_RD_12 | pPt-539905 | H | 24.5 | 3.21 | 9.5 |
|  | B_RD_13 |  |  |  | 4.00 | 12.2 |
|  | B_RD_14 |  |  |  | 4.53 | 13.5 |
| VIII | B_RD_14 | pPt-535666 | P1 | 65.2 | 4.09 | 12.3 |
| IX | B_RD_12 | pPt-471368 | P1 | 73.4 | 3.06 | 9.0 |
| X | B_RD_12 | pPt-653829 | P2 | 15.5 | 4.50 | 13.0 |
| XII | B_RD_12 | pPt-651718 | H | 49.2 | 4.99 | 14.3 |
|  | B_RD_13 |  |  |  | 2.96 | 9.1 |
|  | B_RD_14 |  |  |  | 3.80 | 11.4 |
| V | SCB_RD_12 | pPt-655798 | P2 | 31.6 | 3.55 | 10.4 |
|  | SCB_RD_13 |  |  |  | 7.29 | 21.2 |
|  | SCB_RD_14 |  |  |  | 7.35 | 20.9 |
| VIII | SCB_RD_14 | pPt-535666 | P1 | 65.2 | 3.95 | 11.9 |
| XII | SCB_RD_12 | pPt-651718 | H | 49.2 | 4.72 | 13.6 |
|  | SCB_RD_13 |  |  |  | 3.12 | 9.7 |
|  | SCB_RD_14 |  |  |  | 3.74 | 11.3 |
| I | B_FD_13 | pPt-538190 | P2 | 72.9 | 4.68 | 13.6 |
|  | B_FD_14 |  |  |  | 5.96 | 17.4 |
| V | B_FD_13 | pPt-539905 | H | 24.5 | 3.32 | 9.9 |
|  | B_FD_14 |  |  |  | 5.04 | 14.9 |
| V | SCB_FB_13 | pPt-540393 | H | 32.5 | 6.23 | 17.7 |
|  | SCB_FB_14 |  |  |  | 6.91 | 19.8 |
| I | ED12 | toPt-437458 | H | 75.4 | 5.57 | 16.0 |
|  | ED13 |  |  |  | 4.93 | 14.1 |
|  | ED14 |  |  |  | 5.25 | 15.3 |
| I | ED13 | pPt-538519 | H | 13.7 | 4.40 | 12.7 |
| III | ED14 |  |  | 29.7 | 4.04 | 12.0 |
| V | ED12 | pPt-650647 | H | 26.0 | 4.27 | 12.5 |
|  | ED13 |  |  |  | 9.49 | 25.4 |
|  | ED14 |  |  |  | 4.46 | 13.2 |
| VII | ED13 | pPt-654764  - pPt-653593 | H,P2 | 47.3 | 3.72 | 10.9 |
|  | ED14 |  |  |  | 4.20 | 12.5 |
| VIII | ED12 | pPt-535666 | P1 | 65.2 | 4.47 | 13.1 |
|  | ED13 |  |  |  | 3.17 | 9.3 |
|  | ED14 |  |  |  | 3.26 | 9.8 |
| X | ED12 | pPt-456962 | P2 | 15.6 | 3.24 | 9.7 |
|  | ED14 |  |  |  | 3.03 | 9.2 |
| I | TSC12 | pPt-538418 | H | 75.7 | 8.65 | 23.5 |
|  | TSC13 |  |  |  | 6.91 | 19.2 |
|  | TSC14 |  |  |  | 6.46 | 18.7 |
| III | TSC13 | pPt-456904 | H | 0.0 | 4.58 | 13.2 |
|  | TSC14 |  |  |  | 5.29 | 15.6 |
| III | TSC13 | pPt-472721- pPt-536347 | H | 14.1 | 3.31 | 9.7 |
|  | TSC14 |  |  |  | 3.47 | 10.5 |
| III | TSC13 | pPt-458741 | P2 | 32.4 | 3.70 | 10.8 |
|  | TSC14 |  |  |  | 3.42 | 10.4 |
| V | TSC13 | pPt-652324 | P1 | 25.3 | 5.06 | 15.5 |
| V | TSC13 | pPt-559550 | H | 40.7 | 5.73 | 16.1 |
| IX | TSC12 | pPt-657529- pPt-656452 | H,P2 | 1.0 | 4.54 | 13.1 |
|  | TSC13 |  |  |  | 3.26 | 9.6 |
|  | TSC14 |  |  |  | 3.67 | 11.1 |
| IX | TSC12 | pPt-473072 | H | 13.5 | 4.51 | 13.0 |
|  | TSC14 |  |  |  | 4.41 | 12.4 |
| IX | TSC12 | pPt-471870 | H | 33.6 | 4.19 | 12.2 |
|  | TSC14 |  |  |  | 4.00 | 12.0 |
| X | TSC12 | pPt-652232 | H | 72.2 | 3.10 | 9.1 |
|  | TSC13 |  |  |  | 3.15 | 9.3 |

^a/^ P1- inherited from DG 03-226, P2- inherited from DG 06-5, H- descended from both parents
