# Supplementary material for: Quantitative trait loci for tuber blackspot bruise and enzymatic discoloration susceptibility in diploid potato
Source: Mol Genet Genomics. 2017 Oct 27;293(2):331–42. doi: 10.1007/s00438-017-1387-0 (PMC5854731; doi:10.1007/s00438-017-1387-0)
Supplement: Supplementary file 6 — Supplementary material 6 (DOCX 16 KB) [file 438_2017_1387_MOESM6_ESM.docx]

**Quantitative trait loci for tuber blackspot bruise and enzymatic discoloration susceptibility in diploid potato.**

A. Hara-Skrzypiec, J. Śliwka, H. Jakuczun, E. Zimnoch-Guzowska

Plant Breeding and Acclimatization Institute – National Research Institute, Młochów, Platanowa 19, 05-831Młochów, Poland.

*Corresponding author: Agnieszka Hara-Skrzypiec; a.hara@ihar.edu.pl

**Supplementary Table S4** Physical location of QTL detected using diploid mapping population 11-36 in DM1-3- v4.03 potato genome. QTL for: mean blackspot bruise susceptibility (B_RD ,_ B_FB_), mean starch content- corrected blackspot bruise susceptibility (SCB_RD_,SCB_FB_), mean enzymatic discoloration (ED) and mean tuber starch content (TSC)

| Chromosome | Trait | Significant interval (cM) | Flanking markers | Position of the marker in  DM1-3- v4.03 potato genome | |
| --- | --- | --- | --- | --- | --- |
| I | ED | 13.6-14.7 | pPt-535519- pPt-538519 | | chr01:5175555..5175807 chr01:5175555..5175807 |
|  | B_RD_ | 51.5-86.8 | pPt-472129- pPt-653323 | | chr01:75901446..75902146 chr01:87569573..87570249 |
|  | B_FB_ | 54.1-94.8 | capPt-672450-pPt-653323 | | chr01:75836552..7583685 chr01:87569573..87570250 |
|  | ED | 57.4-108.8 | pPt-537507- AGPaseS | | chr01:76944723..76945653 chr01:86092270..86097270 |
|  | TSC | 43.1-108.8 | pPt-534108- AGPaseS | | chr01:66429633..66429927 chr01:86092270..86097270 |
| III | TSC | 0.0-7.9 | pPt-456904- pPt-654214 | | Unknown chr03:1936639..1937543 |
|  |  | 13.0-15.0 | pPt-559412- pPt-536347 | | chr03:51411154..51411461 chr03:51411154..51411461 |
|  |  | 25.4-32.4 | pPt-539809- pPt-458741 | | Unknown chr03:53173122..53173638 |
|  | ED | 28.7-30.7 | pPt-473371- pPt-540060 | | Unknown chr03:54463195..54463738 |
| V | ED | 11.6-44.1 | pPt-472168- pPt-472014 | | chr05:3538067..3538359 chr05:49096306..49096919 |
|  | B_FB_ | 13.6-43.2 | pPt-472168- toPt-439115 | | chr05:3538067..3538360 chr05:48509730..48510375 |
|  | B_RD_ | 22.2-45.1 | pPt-457569- pPt-472014 | | Unknown chr05:49096306..49096919 |
|  | SCB_RD_ | 13.6-60.1 | pPt-654022- pPt-559591 | | Unknown |
|  | SCB_FB_ | 11.6-47.6 | pPt-472168- pPt-657197 | | chr05:3538067..3538359 Unknown |
|  | TSC | 25.3-25.4 | pPt-652324-pPt459253 | | Unknown  Unknown |
|  |  | 35.9-41.6 | toPt-437417- pPt-652436 | | chr05:32437694..32438115 chr05:47902009..47902334 |
| VII | ED | 37.4-54.3 | pPt-457469- pPt-653593 | | Unknown chr07:52744134..52744569 |
| VIII | ED | 35.3-65.2 | pPt-655631-pPt-535666  pPt-535666 | | chr08:48738148..48738528  Unknown |
|  | B_RD_ | 61.7-65.2 | pPt-457967-pPt-535666 | | Unknown  Unknown |
|  | SCB_RD_ | 61.7-65.2 | pPt-457967-pPt-535666 | | Unknown  Unknown |
| IX | TSC | 0-5.5 | pPt-657529- pPt-656452 | | chr09:39345191..39345874  chr09:39345191..39345874 |
|  |  | 11.3-15.7 | pPt-652856- pPt-539125 | | Unknown  Unknown |
|  |  | 29.6-34.7 | pPt-656184- pPt-540213 | | Unknown  Unknown |
| X | ED | 15.6-23.7 | pPt-533816- pPt-650780 | | chr10:2134921..2135409 Unknown |
|  | TSC | 71.7-72.2 | pPt-473162- pPt-652232 | | Unknown chr10:56308739..56309044 |
| XII | B_RD_ | 42.0-69.6 | pPt-651408- toPt-439901 | | chr12:7849989..7850206 Unknown |
|  | SCB_RD_ | 43.7-72.6 | pPt-473252- pPt-653686 | | Unknown |
